# Subaortic Membrane Papillary Fibroelastoma

**DOI:** 10.1155/2020/2586730

**Published:** 2020-01-09

**Authors:** Robyn Bryde, Kevin Landolfo, Jordan C. Ray, Pragnesh Parikh, Qihui “Jim” Zhai, Carolyn Landolfo

**Affiliations:** ^1^Department of Internal Medicine, Mayo Clinic Florida, 4500 San Pablo Road South, Jacksonville, Florida 32224, USA; ^2^Department of Cardiothoracic Surgery, Mayo Clinic Florida, 4500 San Pablo Road South, Jacksonville, Florida 32224, USA; ^3^Department of Cardiovascular Disease, Mayo Clinic Florida, 4500 San Pablo Road South, Jacksonville, Florida 32224, USA; ^4^Department of Pathology, Mayo Clinic Florida, 4500 San Pablo Road South, Jacksonville, Florida 32224, USA

## Abstract

A 61-year-old male presented for an annual exam and received a transthoracic echocardiogram (TTE) which revealed a mobile mass arising from a subaortic membrane. Further investigations with a transesophageal echocardiogram (TEE) and cardiac computerized tomography angiography (CTA) confirmed the presence of a mobile 9 mm × 3 mm mass on a subaortic membrane. Cardiothoracic surgery was performed with an open operation removing the mass and subaortic membrane. Upon visual inspection, the mass was likened to a sea anemone and immunohistochemical staining performed pathologically confirmed the diagnosis of cardiac papillary fibroelastoma. This case represents the first reported example of a cardiac papillary fibroelastoma (PFE) arising from a subaortic membrane. Although PFEs are benign cardiac tumors, proper identification and consideration for excision of these lesions may be indicated to prevent thromboembolic complications.

## 1. Introduction

Cardiac papillary fibroelastomas (PFEs) ensue as the second most common form of primary cardiac tumors in the adult population and are frequently found incidentally during cardiac imaging [1, 2]. Approximately 85% of PFEs are isolated to valvular surfaces in the following order of frequency: aortic, mitral, tricuspid, and pulmonary valves [1]. The remaining 15% of tumors involve nonvalvular sites, typically left-sided endocardial surfaces. We present a rare case of a PFE arising from a subaortic membrane, which has not been reported previously. The tumor and subaortic membrane were surgically resected without complications.

## 2. Case Report

**Figure 1:**
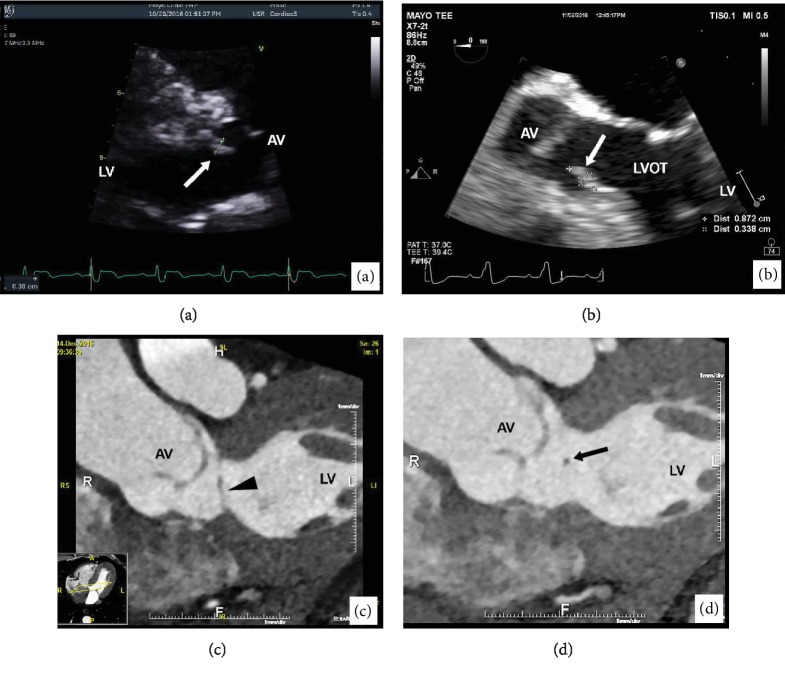
(a) TTE imaging of a subaortic membrane and mass (arrow) on the anterior wall of the LVOT. (b) TEE imaging showing the mass (arrow) in close proximity to the aortic valve. (c) CTA showing a subaortic membrane (arrowhead) with an area of extension, likely representing a mass (arrow) in (d).

A 61-year-old gentleman presented for a health maintenance examination. His medical history was significant for diabetes mellitus type 2, hypertension, hyperlipidemia, and IgM monoclonal gammopathy of unknown significance. He reported no symptoms and maintained an active lifestyle by exercising several times per week. Physical exam findings revealed a BP of 139/81 and heart rate of 70 beats per minute with a regular rate and rhythm and no murmurs or gallop. Other systems were unremarkable, including the absence of systemic symptoms. The patient's baseline ECG showed minor abnormalities. In the setting of chronic hypertension and ECG abnormalities, a TTE was obtained and showed normal left ventricular size and function, but incidentally revealed a subaortic membrane in addition to a highly mobile mass arising from the membrane (Figure 1(a)). Differential diagnosis of the mass included endocardial tumor, thrombus, or endocarditis. Due to the highly mobile nature of the mass, endocardial thrombus was less likely. Additionally, in the absence of clinical evidence of infection, endocarditis was ruled out. The patient was referred to cardiovascular medicine for further investigation of the cardiac mass.

TEE confirmed the presence of a mobile 9 mm × 3 mm mass on the subaortic membrane within the LVOT suspicious for a PFE (Figure 1(b)) (video [Supplementary-material supplementary-material-1]). No Doppler evidence of significant left ventricular outflow tract (LVOT) obstruction was present; however, due to the size, mobility, and location of the mass, cardiothoracic surgery was consulted for surgical removal of the mass.

In preparation for surgery, the patient received a preoperative cardiac CTA which showed an insignificant coronary atherosclerotic plaque and confirmed the presence of the mass on a nonobstructive subaortic membrane 11 mm from the aortic valve (Figures 1(c) and 1(d)).

**Figure 2:**
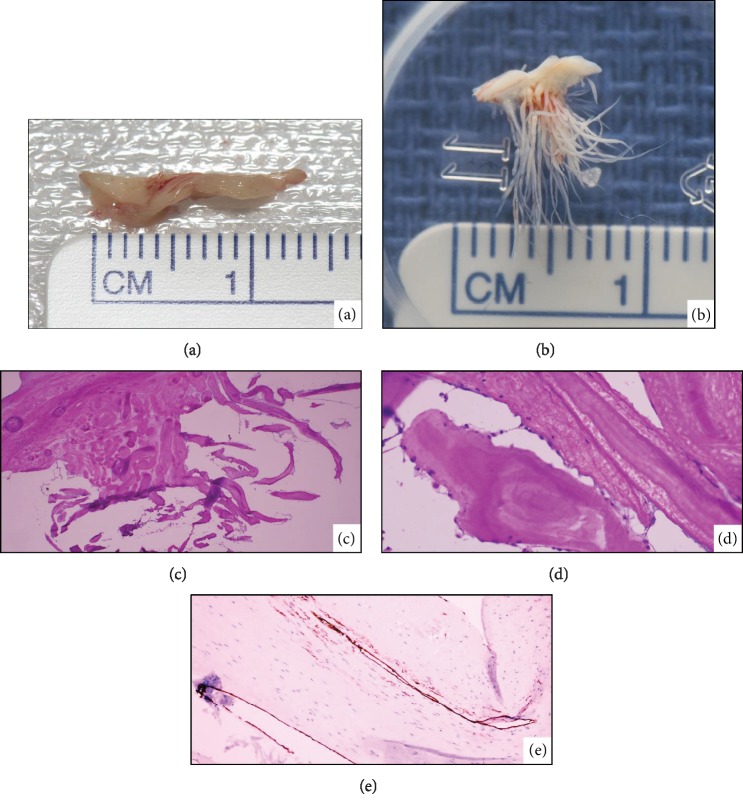
Gross specimens of the resected subaortic membrane (a) and mass (b) which has the classic frond-like appearance after being submerged in water. Histopathology with H&E stain revealing multiple papillae (c) which are avascular and rich in collagen (d) and CD34 staining highlighting the dark single layer of epithelial cells lining the mass.

The operation was performed through a median sternotomy, and the patient was placed on cardiopulmonary bypass. Inspection of the LVOT revealed a multifaceted mass extending from a partial subaortic membrane, the appearance of which was consistent with a papillary fibroelastoma. The mass was excised and the subaortic membrane was dissected to the muscular portion of the septum (Figure 2(a)). The specimen was submerged in water revealing the frond-like appearance which can be likened to a sea anemone (Figure 2(b)). The patient had an uneventful postoperative course.

Histologically, hematoxylin and eosin (H&E) stain of the specimen revealed branching papillae (Figure 2(c)) which were avascular and rich in collagen (Figure 2(d)). CD34 stain highlighted the surface composed of a single layer of endothelial cells (Figure 2(e)). The pathologic examination confirmed the diagnosis of PFE.

## 3. Discussion

PFEs are the second most common primary cardiac tumor after myxomas, accounting for approximately 5% to 15% of all cardiac tumors [1]. Additionally, they are the most common type of valvular tumor and usually arise from the mid portion of the valve [1, 3]. PFEs preferentially involve the aortic, mitral, tricuspid, and least commonly the pulmonary valve. These lesions can be rarely found on endocardial surfaces.

The pathogenesis of PFEs remains controversial with origins related to reactive, hamartomatous, thrombotic, viral, or neoplastic processes [4]. Differential diagnosis includes other cardiac tumors (myxoma, fibroma, and rhabdomyoma), thrombi, vegetations (sterile or septic), calcifications, and Lambl's excrescences. Location, mobility, and patient demographics can be used to further characterize PFEs from other cardiac masses.

After surgical excision, submerging the specimen in water will remove adherent clot and reveal the unique appearance which can be likened to a sea anemone with frond-like arms arising from a pedunculated stalk. Diagnosis is confirmed by immunohistochemical staining with Alcian blue, CD34, and elastin revealing mucopolysaccharide, endothelial, and elastic composition, respectively [2].

Most cases are found in asymptomatic patients as incidental findings during cardiac imaging. Symptomatic patients usually present with complications related to thromboembolic events such as transient ischemic attacks, stroke, chest pain, myocardial infarction, syncope, and blindness. Embolism occurs from either the tumor itself which is soft and flimsy or from adherent clot localized within the fronds. Because PFEs more commonly arise from the higher pressure ventricular surface of valves, they have a higher rate of thromboembolism (34%) in comparison to tumors arising from the lower pressure atrial chambers such as myxomas (24%) [3].

There is general agreement that symptomatic patients be treated with surgical intervention. In asymptomatic patients, surgical intervention should be considered for mobile tumors and/or size > 1 cm [5].

## 4. Conclusion

Cardiac papillary fibroelastomas are rare and benign cardiac tumors with the majority occurring on left-sided cardiac valves. This case presents a novel example of a PFE emanating from a subaortic membrane. Although PFEs typically present as asymptomatic incidental findings on imaging, proper identification is important to prevent thromboembolic events. Treatment with surgical resection tends to be curative and with low risk of recurrence [1].

## Abbreviations

AV: Aortic valve
CTA: Computerized tomography angiography
LV: Left ventricle
LVOT: Left ventricular outflow tract
PFE: Papillary fibroelastoma
TTE: Transthoracic echocardiogram
TEE: Transesophageal echocardiogram.

## Supplementary Materials

Supplementary Materials Video File 1: TEE imaging showing a mobile mass on a subaortic membrane within the LVOT.
